# Different Pharmacokinetics of Tramadol, *O*-Demethyltramadol and *N*-Demethyltramadol in Postoperative Surgical Patients From Those Observed in Medical Patients

**DOI:** 10.3389/fphar.2021.656748

**Published:** 2021-04-15

**Authors:** Nenad Neskovic, Dario Mandic, Saska Marczi, Sonja Skiljic, Gordana Kristek, Hrvoje Vinkovic, Boris Mraovic, Zeljko Debeljak, Slavica Kvolik

**Affiliations:** ^1^ Department of Anesthesiology, Resuscitation and ICU, Osijek University Hospital, Osijek, Croatia; ^2^ Faculty of Medicine, University Josip Juraj Strossmayer, Osijek, Croatia; ^3^ Department of Clinical and Laboratory Diagnostics, Osijek University Hospital, Osijek, Croatia; ^4^ Laboratory for Molecular and HLA Diagnostic, Department of Transfusion Medicine, Osijek University Hospital, Osijek, Croatia; ^5^ University of Missouri, Department of Anesthesiology and Perioperative Medicine, School of Medicine, Columbia, MO, United States

**Keywords:** postoperative analgesia, postoperative pain, inflammation, cholinesterase, CYP2D6, O-demethyltramadol, N-demethyltramadol, tramadol

## Abstract

**Background:** Most studies examining tramadol metabolism have been carried out in non-surgical patients and with oral tramadol. The aim of this study was 1) to measure concentrations of tramadol, *O*-demethyltramadol (ODT), and *N*-demethyltramadol (NDT) in the surgical patients admitted to the intensive care unit (ICU) within the first 24 postoperative hours after intravenous application of tramadol, and 2) to examine the effect of systemic inflammation on tramadol metabolism and postoperative pain.

**Methods:** A prospective observational study was carried out in the surgical ICU in the tertiary hospital. In the group of 47 subsequent patients undergoing major abdominal surgery, pre-operative blood samples were taken for *CYP2D6* polymorphism analysis. Systemic inflammation was assessed based on laboratory and clinical indicators. All patients received 100 mg of tramadol intravenously every 6 h during the first postoperative day. Postoperative pain was assessed before and 30 min after tramadol injections. Tramadol, ODT, and NDT concentrations were determined by high-performance liquid chromatography.

**Results:** *CYP2D6* analysis revealed 2 poor (PM), 22 intermediate (IM), 22 extensive (EM), and 1 ultrafast metabolizer. After a dose of 100 mg of tramadol, t_1/2_ of 4.8 (3.2–7.6) h was observed. There were no differences in tramadol concentration among metabolic phenotypes. The area under the concentration–time curve at the first dose interval (AUC_1-6_) of tramadol was 1,200 (917.9–1944.4) μg ×h ×L^−1^. NDT concentrations in UM were below the limit of quantification until the second dose of tramadol was administrated, while PM had higher NDT concentrations compared to EM and IM. ODT concentrations were higher in EM, compared to IM and PM. ODT AUC_1-6_ was 229.6 (137.7–326.2) μg ×h ×L^−1^ and 95.5 (49.1–204.3) μg ×h ×L^−1^ in EM and IM, respectively (*p* = 0.004). Preoperative cholinesterase activity (ChE) of ≤4244 U L^−1^ was a cut-off value for a prediction of systemic inflammation in an early postoperative period. NDT AUC_1-6_ were significantly higher in patients with low ChE compared with normal ChE patients (*p* = 0.006). Pain measurements have confirmed that sufficient pain control was achieved in all patients after the second tramadol dose, except in the PM.

**Conclusions:** *CYP2D6* polymorphism is a major factor in *O*-demethylation, while systemic inflammation accompanied by low ChE has an important role in the *N*-demethylation of tramadol in postoperative patients. Concentrations of tramadol, ODT, and NDT are lower in surgical patients than previously reported in non-surgical patients.

Clinical Trial Registration: ClinicalTrials.gov, NCT04004481.

## Introduction

Tramadol is a commonly used analgesic in ICUs. It is metabolized in the liver through cytochrome P450 to 11 demethylation products. The most important ones are *O*-demethyltramadol and *N*-demethyltramadol. The CYP2D6 isoenzyme metabolizes tramadol into the active ODT, which is responsible for most of the analgesic effect and has a 200-fold greater affinity for opioid receptors than tramadol (Gillen et al., 2000; Grond and Sablotzki, 2004). *CYP2D6* gene is extremely polymorphic, with over 100 allelic variants (Fulton et al., 2019). The alleles vary between fully functional to completely non-functional, producing thus a variety of metabolic phenotypes: ultrafast (UM), extensive (EM), intermediate, and poor metabolizers (Ganoci et al., 2017; Dagostino et al., 2018). In PMs low amounts of ODT are produced and the analgesic effect of tramadol is markedly reduced. Conversely, UMs have a high concentration of active metabolites and could be at increased risk for toxicity (Smith et al., 2018). *N*-demethylation of tramadol into the inactive NDT is catalyzed by the isoenzyme CYP3A4 and CYP2B6. Although both isoenzymes exhibit gene polymorphism and are susceptible to induction or inhibition by some substrates, no significant differences in tramadol metabolism were observed (Miotto et al., 2017). In addition to the genotype, cytochrome activity is influenced by a number of pathophysiological factors, including proinflammatory cytokines, which reduce cytochrome activity (He et al., 2015). In the work of Tanaka and co-workers, a high level of interleukin (IL)-6 was associated with an increased *N*-demethylation of tramadol (Tanaka et al., 2018).

Surgery causes a physiological inflammatory response consisting of complex metabolic, hemodynamic, hormonal, and immune changes, which ensure wound healing after surgery (Finnerty et al., 2013). Tissue injury causes an increase in proinflammatory cytokines, tumor necrosis factor-alpha (TNF)-alpha, IL-1, and IL-6, as well as anti-inflammatory cytokines, IL-10 (Hsing and Wang, 2015). Under physiological conditions, the pro and anti-inflammatory systems are in equilibrium. Dysregulation of the immune system with excessive activity of the proinflammatory response leads to the development of systemic inflammation (Paruk and Chausse, 2019). Systemic inflammation occurs in more than 40% of patients during hospitalization, and is particularly common in surgical ICUs, where its prevalence is more than 80% (Brun-Buisson, 2000; Churpek et al., 2015). There are numerous biological markers of systemic inflammation, and in routine clinical practice, the most common is the use of C-reactive protein (CRP) and procalcitonin, whose synthesis is induced by IL-6 (Paruk and Chausse, 2019).

In recent years, the role of the cholinergic nervous system in maintaining homeostasis during the inflammatory response has been extensively studied, and a marker of the cholinergic system readily available in everyday clinical practice is plasma cholinesterase activity (ChE). The cholinergic nervous system plays a central role in inflammatory processes and it is an efferent part of the neuroimmunological reflex. The inflammatory response caused by surgical injury stimulates the activity of the parasympathetic nervous system and activates the anti-inflammatory process as part of the nervous control of innate immunity (Tracey, 2010; Zivkovic et al., 2017). The cholinergic anti-inflammatory process is mediated by acetylcholine and acts by inhibiting the production of TNF-alpha and IL-1 and suppressing the activation of nuclear factor-kappa B (Das, 2007). Cholinesterase hydrolyzes acetylcholine and reduces its plasma concentration. The exact mechanism of reduced plasma ChE activity in states of acute inflammation has not yet been completely clarified. The low plasma ChE activity tends to maintain high levels of acetylcholine and enhance the negative feedback of the cholinergic system to acute inflammation. Plasma ChE activity reflects cholinergic, non-nervous activity and neuroimmune interactions. Low plasma ChE would signal an interruption in acetylcholine hydrolysis and disruption of immune homeostasis and is the earliest predictor of systemic inflammation, which changes in plasma even before standard inflammatory biomarkers. (Zivkovic et al., 2016). The aim of this study was to measure plasma concentrations of tramadol, ODT, and NDT in the patients admitted to the surgical ICU after major abdominal surgery. We hypothesized that patients with preoperative low plasma ChE activity as part of systemic inflammation in the early postoperative period would have higher plasma NDT levels and a different ratio of tramadol and its demethylation metabolites.

## Patients and Methods

This prospective observational study recruited patients admitted to the surgical ICU after major abdominal surgery. The study was approved by the by the Ethics Committee of the Osijek University Hospital (No. 12272–7/2017), and all patients included in the study signed informed consent. Major abdominal surgery was defined as an open laparotomy with resection of parts of the digestive system. Exclusion criteria for study was a known allergic reaction to tramadol, tramadol therapy in the last 7 days prior surgery, patient age younger than 18 years old, body mass index (BMI) < 18 kg per m^2^ or >40 kg per m^2^, laparoscopic approach to surgery, and liver or renal failure verified before surgery according to Child-Pugh and Kidney disease Improving Global Guidelines (KDIGO) criteria (Durand and Valla, 2005; Khwaja, 2012). Also, patients on chronic therapy with cimetidine, paroxetine, pimozide, metoclopramide, amiodarone, olanzapine, chlorpromazine, fluphenazine, haloperidol, thioridazine, risperidone and clozapine were not included in the study as these drugs inhibit the activity of CYP2D6 enzyme (Bahar et al., 2017).

Pre-operative blood samples were taken in all patients for white blood count (WBC) (Sysmex XN-2000, Sysmex, Kobe, Japan), procalcitonin (PCT) (Roche Cobas E 411, Roche Diagnostics GmbH, Mannheim, Germany), c-reactive protein (CRP), lactate level, urea, creatinine, aspartate aminotransferase, alanine aminotransferase, gamma-glutamyltransferase, total bilirubin, albumin, plasma ChE activity (Beckman Coulter AU680, Beckman Coulter, Brea, CA, USA), and arterial blood gas analysis (Radiometer ABL800 FLEX, Radiometer Medical A/S, Bronshoj, Denmark), according to the manufacturer’s instructions. *CYP2D6* gene polymorphism was determined in all patients.

All patients had general anesthesia with sevoflurane in oxygen and air with standard intraoperative monitoring. An arterial catheter was placed in all patients. Induction was with either propofol or etomidate and rocuronium or succinylcholine depending on patients' hemodynamic status and the procedure urgency. After the surgery, patients were admitted to the ICU, where mechanical ventilation was continued until extubation, as well as monitoring of vital parameters. The development of systemic inflammation during the first 24 h was defined as the presence of at least two of four major criteria: tachycardia >90 beats minute^−1^, fever >38°C or hypothermia <36°C, WBC >12.000/mm^3^ or <4.000/mm^3^, and PaCO_2_ < 4.3 kPa (Marik and Taeb, 2017). Preoperative CRP >50 mg L^−1^ and PCT >0.5 μg L^−1^ were also considered systemic inflammation (Castelli et al., 2004).

### Postoperative Analgesia and Pain Assessment

After ICU admission patients received 100 mg of tramadol (Tramal, Stada Arzneimittel AG, Germany) in 50 ml of 0.9% saline as intravenous (IV) bolus over 10 min at 0^th^, 6^th^, 12th, 18th, and 24th postoperative hours. Paracetamol (Paracetamol B. Braun, B. Braun Medical S.A. Spain) 1 g IV was given between these injections every 6 h. The pain was assessed at 5-time points, corresponding with tramadol administration, before and 30 min after the tramadol dose. In awake patients, verbal Numeric Rating Scale (NRS) values of 3 or less were considered to have adequate analgesia (Gerbershagen et al., 2011). Morphine 2 mg IV bolus was administered in case of insufficient analgesia after tramadol and at the request of the patient during the day. Total morphine consumption was recorded. In addition, postoperative nausea and/or vomiting (PONV) were recorded within a half-hour after tramadol administration.

### Determination of Plasma Concentration of Tramadol and Metabolites

One, two, and four hours after the first dose and before the second, third, and fifth doses of tramadol, arterial blood samples were taken for analyzes of plasma concentrations of tramadol, ODT, and NDT. The analyses were performed by high-performance liquid chromatography (HPLC) on a Shimadzu Nexera XR device (Shimadzu Corporation, Kyoto, Japan). A reversed-phase chromatography system with fluorescence detection (excitation wavelength 200, emission 301 nm) was used. The separation was performed on an Agilent Zorbax SB-C8 column (Agilent Technologies, Santa Clara, CA, United States), 3.5 μm, 4.6 × 150 mm, using a mobile phase consisting of methanol and 1.5 mM H3PO4, pH 2.5 (ratio 19:81, both from Merck, Darmstadt, Germany). Limits of quantitation of tramadol, ODT, and NDT concentrations were 3.9 μg L^−1^, 4.52 μg L^−1^, and 3.52 μg L^−1^, respectively. The area under the concentration-time curve (AUC) was calculated using the linear trapezoidal method (Pruessner et al., 2003). Plasma half-life (t_1/2_) of tramadol was calculated as 0.693/elimination rate constant (β). This constant was estimated as the absolute value of the slope of a square linear regression of the logarithmic plasma concentration-time curve, at tramadol first dose interval.

### *CYP2D6* Polymorphism Analysis and Metabolic Phenotype Classification

A commercial High Pure PCR Template Preparation Kit was used to isolate the patients’ genomic DNA according to the manufacturer’s instructions (Roche Diagnostics, Mannheim, Germany). To determine the allelic variants of CYP2D6*3 (rs35742686), CYP2D6*4 (rs3892097), and CYP2D6*5 (whole gene deletion), the commercial LightMix Kit CYP2D6 *3 *4 and *5/*5 was used according to the manufacturer's instructions (TibMolbiol, Berlin, Germany). Commercial kits “CYP2D6 TaqMan Copy Number Assay” and “TaqMan Copy Number Reference Assay” were used to determine duplication/amplification and copy number (CN) of the *CYP2D6* gene (CYP2D6*1xN) according to the manufacturer’s instructions (Applied Biosystems, Waltham, Massachusetts, United States). The *CYP2D6* allelic variants determination and copy number determination were performed on the LightCycler 480II real-time PCR system (Roche Life Sciences, Mannheim, Germany). Relative quantification (RQ) of *CYP2D6* gene copy numbers in each test sample was performed using the comparative C_T_ (ΔΔC_T_) method (Livak and Schmittgen, 2001). Each allele was assigned an activity score (AS) depending on the known genotype activity (Gaedigk et al., 2008). According to current Clinical Pharmacogenetics Implementation Consortium (CPIC) recommendations, patients were categorized into metabolic phenotypes (Caudle et al., 2020).

### Statistical Analysis

Numerical data are presented by medians and interquartile ranges, and categorical data by absolute and relative frequencies. The normality of the distribution was tested by the Shapiro-Wilk’s test. Differences between numerical data were tested with the Mann-Whitney *U* test, and between categorical data with Fisher exact test. Friedman’s test was used to detect the differences in the concentration of tramadol and metabolites in the six measurement points within the same group. Wilcoxon test was used to analyze pain perception in five paired measurements. Multivariate logistic regression was applied to perform the systemic inflammatory prediction model. Differences between predictors were calculated by area under the receiver operating characteristic (ROC) curve with a 95% confidence interval (CI). A minimum of 40 patients is required to observe differences in the concentrations of tramadol and metabolites between the intermediate and extensive metabolizers with a significance level of 0.05 and a test strength of 80% (with an effect of d = 0.35). All *p* values are two-sided. The significance level was set to Alpha <0.05. The statistical analysis was performed using MedCalc Statistical Software (MedCalc Software Ltd., Ostend, Belgium; https://www.medcalc.org; 2020) version 19.1.7, and IBM SPSS (SPSS Inc. Armonk, NY: IBM Corp.) Version 26.0. Patients with missing data were excluded from analyses. The number of patients included is shown per each analysis.

## Results

Between January 2019 and January 2020, 50 consecutive patients were eligible for the study. Three patients were excluded from the study due to technical errors in the ICU protocol or errors in blood sampling for analysis, and 47 patients were analyzed. The demographic characteristics of the patients are presented in Table 1. According to *CYP2D6* genotype, 2 (4%) were PM, 22 (47%) IM, 22 (47%) EM, and 1 (2%) patient was UM. *CYP2D6* diplotype and metabolic phenotype are shown in Table 2.

**TABLE 1:** Demographic characteristics of patients and *CYP2D6* phenotype. Data are presented as median (interquartile range), or N and ratio (%). BMI, body mass index; ASA, American Society of Anesthesiologists Physical Status Classification System.

Demographic characteristic	Number of patients
Sex (male/female)	30 (64)/17 (36)
Age (years)	67 (59–73)
BMI (kg/m^2^)	26.1 (22.9–28.7)
ASA status II III IV V	11 (23) 27 (58) 8 (17) 1 (2)
Elective/emergency surgery	36 (77)/11 (23)
Metabolic phenotype Poor Intermediate Extensive Ultrafast	2 (4.3) 22 (46.8) 22 (46.8) 1 (2)

**TABLE 2:** *CYP2D6* diplotype, assigned activity score, and metabolic phenotype. PM, poor metabolizer; IM, intermediate metabolizer; EM, extensive metabolizers; UM, ultrafast metabolizers.

Dyplotype	Activity score	Metabolic phenotype (n)
*1/*4	1.0	IM (16)
*1/*1	2.0	EM (22)
*1/*4xN	1.0	IM (3)
*4/*4	0.0	PM (2)
*1/*3	1.0	IM (2)
*1/*1xN	3.0	UM (1)
*1/*5	1.0	IM (1)

### Postoperative Concentrations of Tramadol, ODT and NDT

**FIGURE 1:**
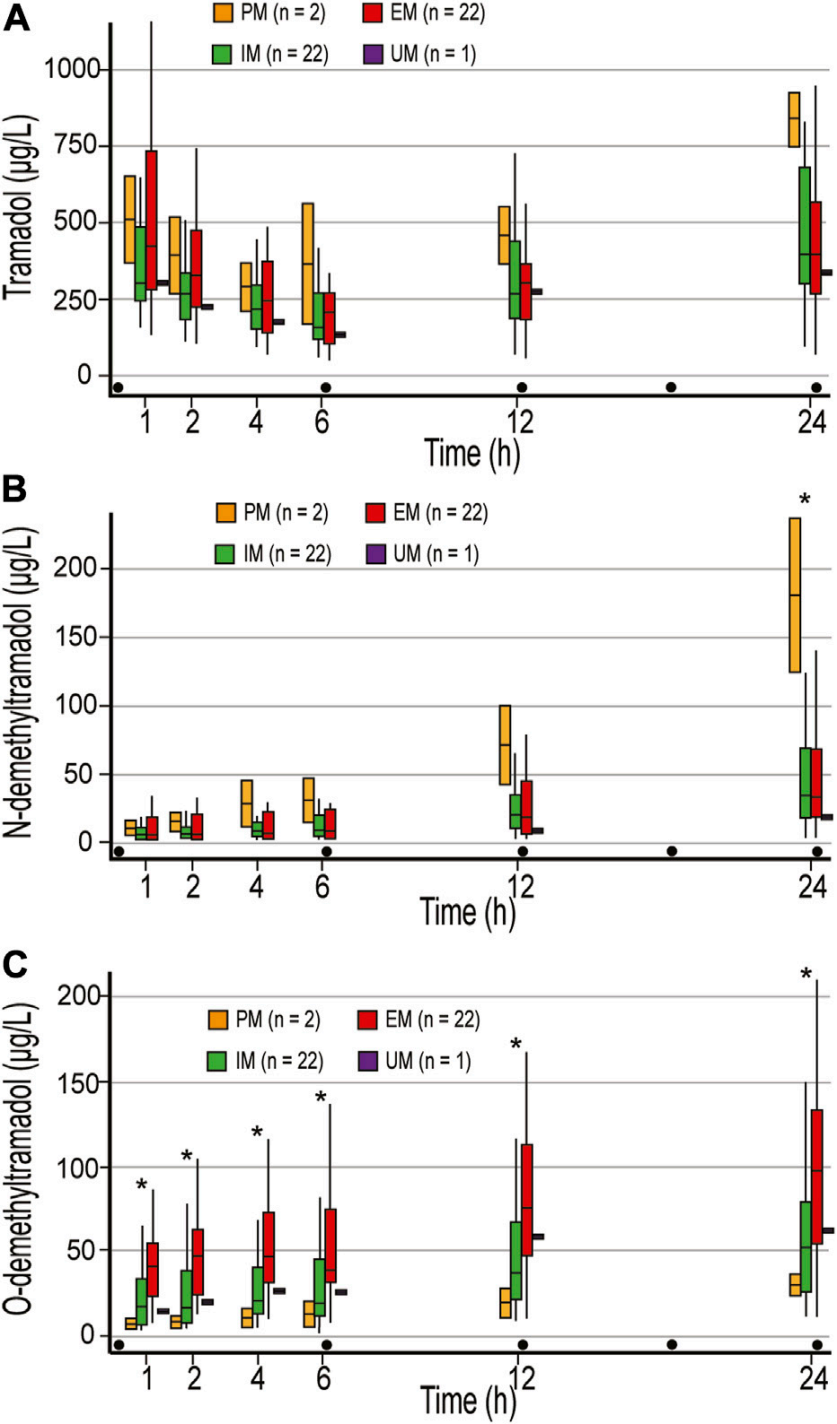
Concentrations of tramadol **(A)**, N-demethyltramadol **(B)**, and O-demethyltramadol **(C)** in the first 24 postoperative hours. Concentrations were measured 1, 2, 4 h after the first dose of 100 mg tramadol iv, and just before the second (time point 6 h), third (time point 12 h), and fifth (time point 24 h) doses of tramadol. PM, poor metabolizer; IM, intermediate metabolizer; EM, extensive metabolizer; UM, ultrafast metabolizers; Dot, tramadol 100 mg IV injections; * statistically significant differences (Mann-Whitney U test) between PM and EM/IM **(B)**, and EM and IM/PM **(C)**.

There were no differences in tramadol concentrations between the different metabolic phenotypes (Figure 1A). Tramadol t_1/2_ of 4.8 (3.2–7.6) h was observed. and the calculated first dose interval AUC (AUC_1-6_) was 1,200.7 (917.9–1944.4) μg ×h ×L^−1^. After 24 h and 400 mg of tramadol, the highest tramadol concentration of 837 μg L^−1^ was measured in PMs.

There were no differences in NDT concentrations between EM and IM, and calculated NDT AUC after 400 mg of tramadol (AUC_1-24_) were 439.7 (201.9–1,061.5) and 474.5 (257–933.8) μg ×h ×L^−1^, respectively. NDT concentrations were higher in PM compared to EM and IM in all measurements, and a statistically significant difference was reached in the last measurement (Figure 1B). One patient who was categorized as UM had NDT concentrations below the limit of quantification for NDT (3.52 μg L^−1^) until the second dose of tramadol was administered (Figure 1B), and had an unexpectedly low concentration of ODT, with maximum of 61.8 μg L^−1^ after 400 mg of tramadol (Figure 1C). Higher concentrations of ODT in EM compared with PM and IM were measured in all measurement points (*p* < 0.05) (Figure 1C). After 400 mg of tramadol, calculated ODT AUC_1-24_ were 435.2 μg ×h ×L^−1^, 784.9 (469.1–1,558.1) μg ×h ×L^−1^, and 1,697.2 (930.6–2,688.7) μg ×h ×L^−1^ in PM, IM, and EM, respectively. As expected, the metabolic ratio (MR) of ODT/tramadol was significantly higher in all measurements in EM compared to IM and PM and was 0.08–0.24, 0.05–0.1, and 0.01–0.03, respectively (*p* < 0.05).

### Systemic Inflammation and Tramadol Metabolism

Due to the important influence of *CYP2D6* polymorphism on ODT and NDT concentrations, and due to the small number of PMs and UMs, the influence of systemic inflammation on tramadol metabolism was analyzed only in EM and IM patients. Based on the clinical and laboratory measurements, postoperative systemic inflammation was confirmed in 17 patients. ROC analysis showed that both preoperative plasma ChE activity and CRP were good predictors of systemic inflammation in the early postoperative period, with a cut-off value in prediction of systemic inflammation of ≤4244 U L^−1,^ and >54.2 mg L^−1^, respectively (Table 3).

**TABLE 3:** ROC curve parameters for systemic inflammation prediction in extensive and intermediate metabolizers. CRP, c-reactive protein; ChE,cholinesterase; AUC, area under the curve; CI, confidence interval; Y,Youden’s index.

	AUC	95% CI	Sensitivity (%)	Specificity (%)	Cut-off	Y	P
CRP	0.756	0.6–0.87	64.7	92.6	>54.2 mg L^−1^	0.57	0.005
ChE	0.762	0.57–0.89	70.6	76.9	≤4244 U L^−1^	0.48	0.001

A subgroup of 18 (41%) patients had low ChE activity, i.e., ≤4244 U L^−1^ (low ChE group—LChE), while 25 (57%) of them are classified as normal ChE group—NChE. Preoperative ChE activity was not registered in one patient who was excluded from analyses.

LChE patients are significantly more likely to have emergency surgery (OR 30, *p* < 0.001) and fulfill the criteria for systemic inflammation within the first 24 h of the ICU admission (OR 8.0, *p* = 0.003). They also had significantly lower red blood cell count (RBC) and albumin levels, with higher pre-operative values of inflammatory parameters and urea **(** Table 4). In comparison to NChE patients, LChE patients spent more time on ventilator during ICU stay, 262.5 (115–746.2) minutes vs. 125 (62.5–255) minutes (*p* = 0.03), spent more days in the ICU, 1.5 (1–6) days vs. 1 (1–1) day, *p* = 0.01), and had longer hospitalization, 12.5 (10–19.5) days vs. 10 (7–14) days, *p* = 0.04).

**TABLE 4:** A comparison of patient characteristics according to preoperative cholinesterase activity. Data are presented as median (interquartile range), or (N) and ratio (%). WBC, white blood cells; RBC, red blood cells; CRP, c-reactive protein; PCT, procalcitonin; AST, aspartate aminotransferase; ALT, alanine aminotransferase; GGT, gamma-glutamyltransferase; PT, prothrombin time; ChE, cholinesterase; NChE, ChE >4244 U L^−1^; LChE, ChE ≤4244 U L^−1^. *Mann-Whitney *U* test for continuous, and Fisher's exact test for categorical variables.

	Median (IQR) or N (%)		P*
	NChE (n = 25)	LChE (n = 18)	
Age (years)	66 (58–72.5)	68 (60.5–78)	0.29
Metabolic phenotype (IM/EM)	12 (48%)/13(52%)	9 (50%)/9 (50%)	>0.99
BMI	26.3 (23.6–29.4)	24.2 (19.4–27.8)	0.04
WBC (x10^3^ L^−1^)	5.4 (4.5–7.8)	9.8 (6.5–12.2)	0.01
RBC (x10^9^ L^−1^)	3.9 (3.6–4.1)	3.4 (3.1–3.5)	<0.001
Hemoglobin (g L^−1^)	113 (100–128)	102 (89.2–106.2)	0.03
Platelets (x10^9^ L^−1^)	187 (159–241)	233 (179–353)	0.01
Urea (mmol L^−1^)	3.9 (3.4–5.1)	7.4 (5.1–15.9)	0.02
Creatinine (μmol L^−1^)	66 (55–80)	80 (47–152.5)	0.49
CRP (mg L^−1^)	4 (1.8–37.8)	47 (11.5–187.9)	0.005
PCT (μg L^−1^)	0.06 (0.03–0.08)	0.14 (0.07–4.42)	0.002
AST (U L^−1^)	19 (13.5–32.5)	26 (16.2–46)	0.22
ALT (U L^−1^)	15 (11–26)	19 (10–25)	0.73
GGT (U L^−1^)	17 (13–38.5)	23.5 (12–99.5)	0.64
Bilirubin (μmol L^−1^)	11 (9–15)	13 (6,7–17,2)	0.72
Albumin (g L^−1^)	32.1 (30.4–35.5)	25.1 (23–29.1)	<0.001
PT (Inr)	1.1 (1.04–1.18)	1.1 (1–1.3)	0.43
ChE (U L^−1^)	6,171 (4,777–6,635)	3,230 (2,837.5–2,766.5)	<0.001
Emergency surgery	1 (4%)	10 (55.6%)	<0.001
Systemic inflammation	5 (20%)	12 (66.7%)	0.004

There were no differences in tramadol concentrations between the LChE and NChE groups. First dose interval AUC (AUC_1-6_) were 1,521.1 (962.1–2,402.8) μg ×h ×L^−1^, and 1,186.9 (797.1–1,646.8) μg ×h ×L^−1^, respectively.

**FIGURE 2:**
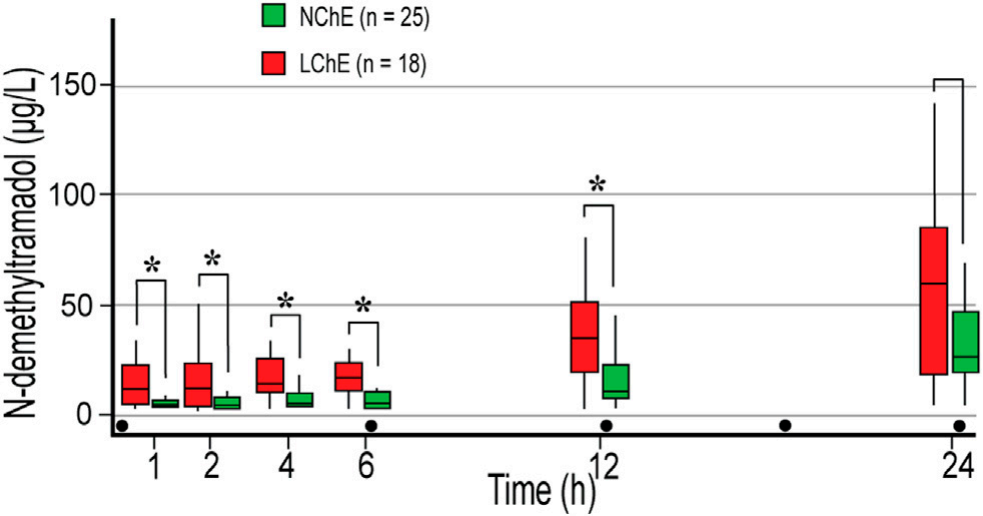
The concentration of *N*-demethyltramadol in the first 24 postoperative hours depending on preoperative plasma cholinesterase activity in extensive and intermediate metabolizers. Concentrations were measured 1, 2, 4 h after the first dose of 100 mg tramadol iv, and before the second (time point 6 h), third (time point 12 h), and fifth (time point 24 h) doses of tramadol. LChE, cholinesterase ≤4244 U L^−1^; NChE, cholinesterase >4244 U L^−1^; dot, tramadol 100 mg IV injections; * statistically significant differences (Mann-Whitney *U* test) between LChE and NChE group.

A correlation analysis confirmed statistically negative correlation between ChE and NDT in the measurement points 1 – 4 (*p* < 0.05). NDT levels were significantly higher in LChE patients than in NChE patients (Figure 2). Calculated NDT AUC_1-24_ were 793 (397.2–1,325.3) μg ×h ×L^−1^, and 357.8 (198.8–527.6) μg ×h ×L^−1^, in LChE and NChE, respectively (*p* = 0.02). Similar observations were made in emergency surgery patients, who had a higher concentration of NDT in the first three measurements. They had about three-time higher NDT in first measurement in compared to elective surgical patients, 14.9 (5.7–27.3) μg L^−1^, vs. 3.9 (3.5–7) μg L^−1^ (*p* = 0.002). Calculated NDT AUC_1-6_ was 73.7 (37.5–134.3) μg ×h ×L^−1^, and 29.2 (19.7–62.8) μg ×h ×L^−1^, in emergency and elective surgery patients, respectively (*p* = 0.02). Patients with developed signs of systemic inflammation in the postoperative period also had higher concentrations of NDT, but only in the first measurement (8 (4.7–17.7′) μg ×L^−1^, vs. 3.7 (3.5–6.5) μg×L^−1^, *p* = 0.02).

MR NDT/tramadol was higher in all measurements in the LChE group compared to the NChE, and statistically significant difference was achieved in the last measurement with median of 0.15 in LChE and 0.06 in NChE group, *p* = 0.03.

In our sample of patients, we did not observe significant differences in ODT concentrations with respect to ChE activity or clinical signs of systemic inflammation. However, the genetically determined difference in MR ODT/tramadol and ODT concentrations between EM and IM (Supplementary Figure S1A) was lost in all patients within the LChE group (Supplementary Figure S1B **)** and in patients who had developed postoperative systemic inflammation (Supplementary Figure S1C).

### Postoperative Analgesia and Tramadol Side Effects

**FIGURE 3:**
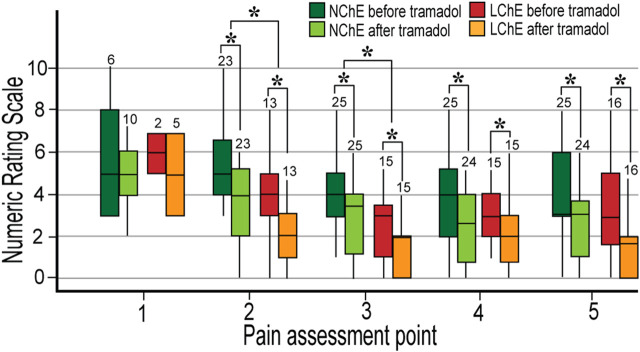
Postoperative pain in the first 24 postoperative hours in the patients with normal and low preoperative plasma cholinesterase activity. Normal metabolizers (EM and IM) were analyzed. The pain was measured five times in 24 h, before and 30 min after 100 mg of tramadol. The numbers of participants are written above the boxplot. LChE: cholinesterase ≤4244 U L^−1^; NChE, cholinesterase >4244 U L^−1^; NRS, Numeric Rating Scale; *statistically significant differences in NRS between LChE and NChE group (upper cluster—Mann-Whitney *U* test) and in NRS before and after tramadol within the same group (lower cluster–Wilcoxon test).

The Wilcoxon paired test showed that tramadol provided effective pain relief measured half an hour after administration of the second dose in all patients, except in PM. There was no difference in the analgesic effect of tramadol regarding the systemic inflammation or preoperative ChE activity (Figure 3).

LChE patients received a lower dose of opioids during surgery (calculated as morphine milligram equivalents) compared to NChE patients, 50 (39–70) mg vs. 79 (61.2–94.7) mg, *p* < 0.001. They still had lower pain reported in the postoperative period at 2^nd^ and 3^rd^ pain assessments, before and after tramadol administration (Figure 3). There was no difference in postoperative morphine consumption between the LChE and NChE groups.

In our sample of patients, pain relief was not in the correlation with tramadol and ODT plasma levels, while NDT concentrations, conversely were in negative correlation with pain in the 4^th^ measurement, Rho = –0.536 (*p* < 0.001). Lower NDT concentrations were registered in the patients who had inadequate pain relief (NRS ≥4) at the 2^nd^ and 3^rd^ pain assessment point, compared to patients with good analgesia, 3.5 μg L^−1^ and 8.1 μg L^−1^ vs. 12.8 and 28.3 μg L^−1^, *p* = 0.001, respectively.

In the first 24 postoperative hours, PONV was observed more often in EM (12 out of 22 patients) than in IM (4 out of 22 patients), *p* = 0.03.

## Discussion

The result of this study showed that concentrations of tramadol, ODT, and NDT are lower in surgical ICU patients than observed earlier in medical patients and healthy volunteers. Data on the concentrations of tramadol and its metabolites available in the literature were obtained mostly on non-surgical patients or healthy volunteers, and the concentrations were measured after a single dose of tramadol. Kirchheiner et al. administered a single oral dose of 100 mg tramadol and measured tramadol C_max_ and ODT C_max_ of 208 μg L^−1^ and 106 μg L^−1^, respectively (Kirchheiner et al., 2008). Ardakani et al. measured tramadol C_max_ of 314.4 μg L^−1^, ODT 88.6 μg L^−1^, and NDT 33.4 μg L^−1^ in women after a single dose of 100 mg tramadol (Ardakani and Rouini, 2007). However, the *CYP2D6* gene polymorphism was not examined in the latter study. Approximately similar concentrations of tramadol, ODT, and NDT were achieved in our subjects only after repeated IV doses of tramadol. ODT concentrations 4 h after tramadol injection in EM were two times lower than the concentrations achieved after only one oral dose of tramadol in healthy subjects in a study by Kirchheiner et al. (Kirchheiner et al., 2008).

Using patient-controlled analgesia, Lehmann et al. concluded that the minimum effective plasma concentrations of tramadol and ODT were 287 μg L^−1^ and 36.2 μg L^−1^, respectively (Lehmann et al., 1990). This level of tramadol was reached in most of our subjects, but this level of ODT was achieved in the first measurement only in EM, while IM reached this ODT level only in the last measurement. Nevertheless, tramadol analgesia was effective in both groups of patients. The metabolic ratio of ODT/tramadol in most studies was about 0.3 (Nobilis et al., 2002; Grond and Sablotzki, 2004; Siepsiak-Połom et al., 2019), while in our IM and EM patients after 2 h it was only 0.07 and 0.16, respectively. The ratio of 0.2 was reached after the 2^nd^ and 3^rd^ doses of tramadol only in EM patients.

The diversity of previously conducted studies is the reason that the data obtained in our study can only be partially compared with them. The comparison with previous studies is difficult because many researchers have not determined the polymorphism of the *CYP2D6* gene. However, the lower concentrations measured in surgical patients in our study differed significantly from the concentrations in medical patients and healthy volunteers (Ardakani and Rouini, 2007; Stamer et al., 2007; Kirchheiner et al., 2008; Lu et al., 2020).

Following the major surgical procedure and high-volume resuscitation in the first 24 postoperative hours, the lower plasma concentrations of tramadol, ODT, and NDT measured in our patients are probably associated with increased volume of distribution. The higher volume of distribution in postoperative patients is associated with hyperpermeability of vascular endothelium which occurs early during inflammation and in the postoperative period due to glycocalyx damage (Uchimido et al., 2019). Endothelial damage and impaired vascular permeability combined with volume resuscitation can significantly increase the volume of drug distribution with a consequent decrease in their plasma concentration (Charlton and Thompson, 2019).

The differences in the concentration of tramadol and its metabolites, as observed in or study, may differ from those reported in other studies; this could be due to different patient populations, as well as due to potentially different approaches to the analytical method used. Our HPLC method for the determination of tramadol concentration is standardized, but small differences in the method and thus in the results may exist among different researchers. Further research (with a control group of non-surgical patients, analyzed with the same analytical method) is needed for a more definite conclusion.

The enzyme cholinesterase in our study significantly correlated with the development of systemic inflammation, and with changes in the concentrations of NDT. Zivkovic et al. have observed that the plasma activity of ChE after trauma-induced systemic inflammation decreases before standard proinflammatory biomarkers began to increase, and confirmed its importance in the early diagnosis of systemic inflammation, and in predicting morbidity and mortality (Zivkovic et al., 2016; Zivkovic et al., 2019). Li et al. demonstrated that low ChE activity at the time of hospitalization was an independent risk factor for death in patients with ischemic stroke. Patients with low ChE activity had higher CRP values, lower albumin, were more often anemic, and had increased mortality and longer hospital stays, as observed in our patients (Li et al., 2020).

Studies in healthy individuals have confirmed that in PMs, due to genetically reduced CYP2D6 activity, tramadol metabolism is predominantly redirected (metabolic switch) to *N*-demethylation (Quetglas et al., 2007; Haage et al., 2018). Our study suggests that a similar metabolic switch may also occur during systemic inflammation with low ChE activity.

To date, no study of tramadol metabolism has been conducted in surgical patients with signs of acute systemic inflammation, and this is the first study to include both elective and emergency patients with inflammatory conditions. Like previous studies, this one confirmed that urgent surgical patients with clinical signs of systemic inflammation were more likely to have low ChE activity (Becher et al., 2012; Smajic et al., 2018).

Surgery and acute inflammation reduced CYP3A4 enzyme activity in the study conducted by Haas et al. in postoperative patients (Haas et al., 2003). This inhibition can lead to changes in the metabolism of drugs that are catalyzed by this enzyme. Proinflammatory cytokines are thought to act indirectly on cytochromes, reducing the expression of transcription factors such as HNF4- α (hepatocyte nuclear factor 4—α) which is responsible for the transcription of the *CYP2D6* gene (He et al., 2015). However, CYP2B6, important in *N*-demethylation of tramadol in NDT, in contrast to CYP2D6 and CYP3A4 enzymes, shows a different response to proinflammatory cytokines. Thus, for example, IL-1, which is a potent inhibitor of the CYP3A4 enzyme, has no influence on CYP2B6. Transforming growth factor-beta (TGF-β), which is secreted from hepatocytes during stress and inflammation, induces CYP2B6 enzyme activity almost two and half times, which was not observed with CYP3A4 and CYP2D6 (Aitken and Morgan, 2007; Babeu and Boudreau, 2014). Unhindered CYP2B6 pathway may be a reason for higher NDT concentration in emergency patients and patients with low ChE activity.

A complex combination of genetic factors, the effect of cytokines on cytochromes, ODT transporters on hepatocytes (Vee et al., 2009; Tzvetkov et al., 2011), and altered volume of distribution may be the reason for the altered ODT concentrations in postsurgical patients. Also, the same factors may have been the cause of the loss of genetically determined differences in ODT concentrations in the group of patients with systemic inflammation. Although the sample of patients in this analysis was small, this observation requires attention and study in a larger number of subjects. All of these factors may influence the level of pain reported, and the effectiveness of drugs used.

Patients with acute inflammation and low pre-operative ChE with altered concentrations of metabolites had a lower expression of pain at all measurement points compared with those having normal ChE. This can be partly explained by brain dysfunction that may co-exist with systemic inflammation, as observed by McGrane and co-workers. The authors found that high values of proinflammatory biomarkers are a good predictor of delirium and coma during critical illness. They have observed that systemic inflammation is important in the pathophysiology of acute brain dysfunction in critically ill patients (McGrane et al., 2011). It should be emphasized here that higher concentrations of inactive NDT metabolite measured in the low ChE patients are not the cause of decreased pain reported. Such higher concentrations are merely a consequence of systemic inflammation that alters both the pain perception and the tramadol metabolism (McGrane et al., 2011; Uchimido et al., 2019).

With the introduction of fast-tracking within the Enhanced Recovery After Surgery protocols (Grant et al., 2017), it is expected that an increasing number of elective patients will bypass the ICUs. At the same time, an increasing proportion of ICU patients will likely be elderly, with numerous comorbidities and emergencies. In these patients, inflammation will be more common, as well as changes in drug metabolism.

The inability to compare pain and PONV in all study patients is a drawback of this study. Predominantly emergency patients who were longer drowsy and were extubated a few hours after ICU admission were not included in the pain analysis at initial pain assessments. It is possible that the altered consciousness in these patients was influenced by systemic inflammation, as mentioned earlier. Furthermore, we have not determined the tramadol enantiomers and their metabolites. The pharmacokinetics of tramadol and its metabolites are known to be stereoselective (Quetglas et al., 2007) (+) ODT is a more potent analgesic than the (−) ODT enantiomer (Grond et al., 1999). It is not yet known how systemic inflammation affects the synthesis of different enantiomers whose ratios can be altered in postoperative patients.

Personalized medicine strives to individualize therapy and determine treatment of patients based on genotype to increase treatment effectiveness with the lowest risk of adverse reactions (Caudle et al., 2014). Our and numerous other studies have confirmed that cytochrome activity is strongly influenced by genetic and pathophysiological factors, one of which is systemic inflammation. This study showed that ChE correlates with systemic inflammation, in addition to affecting tramadol metabolism, also alters pain perception. Consequently, it seems reasonable to conclude that inflammation is indeed a covert threat to effective genotype-based therapy (Shah, 2017).

In conclusion, our study in postoperative surgical patients has confirmed that the *O*-demethylation of tramadol is predominantly influenced by the *CYP2D6* polymorphism, whereas *N*-demethylation is under the strong influence of systemic inflammation. Systemic inflammation also changes the perception of pain, and future studies should confirm whether and how the dose of tramadol should be changed in patients with systemic inflammation.

## Data Availability

The original contributions presented in the study are included in the article/Supplementary Material, further inquiries can be directed to the corresponding authors.
