# Corrigendum: Different pharmacokinetics of tramadol, *O*-demethyltramadol and *N*-demethyltramadol in postoperative surgical patients from those observed in medical patients

**DOI:** 10.3389/fphar.2023.1250698

**Published:** 2023-08-22

**Authors:** Nenad Neskovic, Dario Mandic, Saska Marczi, Sonja Skiljic, Gordana Kristek, Hrvoje Vinkovic, Boris Mraovic, Zeljko Debeljak, Slavica Kvolik

**Affiliations:** ^1^ Department of Anesthesiology, Resuscitation and ICU, Osijek University Hospital, Osijek, Croatia; ^2^ Faculty of Medicine, University Josip Juraj Strossmayer, Osijek, Croatia; ^3^ Department of Clinical and Laboratory Diagnostics, Osijek University Hospital, Osijek, Croatia; ^4^ Laboratory for Molecular and HLA Diagnostic, Department of Transfusion Medicine, Osijek University Hospital, Osijek, Croatia; ^5^ University of Missouri, Department of Anesthesiology and Perioperative Medicine, School of Medicine, Columbia, MO, United States

**Keywords:** postoperative analgesia, postoperative pain, inflammation, cholinesterase, CYP2D6, O-demethyltramadol, N-demethyltramadol, tramadol

In the published article, there was an error in Figure 1 as published. The Figures 1B, C were incorrect and appeared inverted in the figure caption.

**FIGURE 1:**
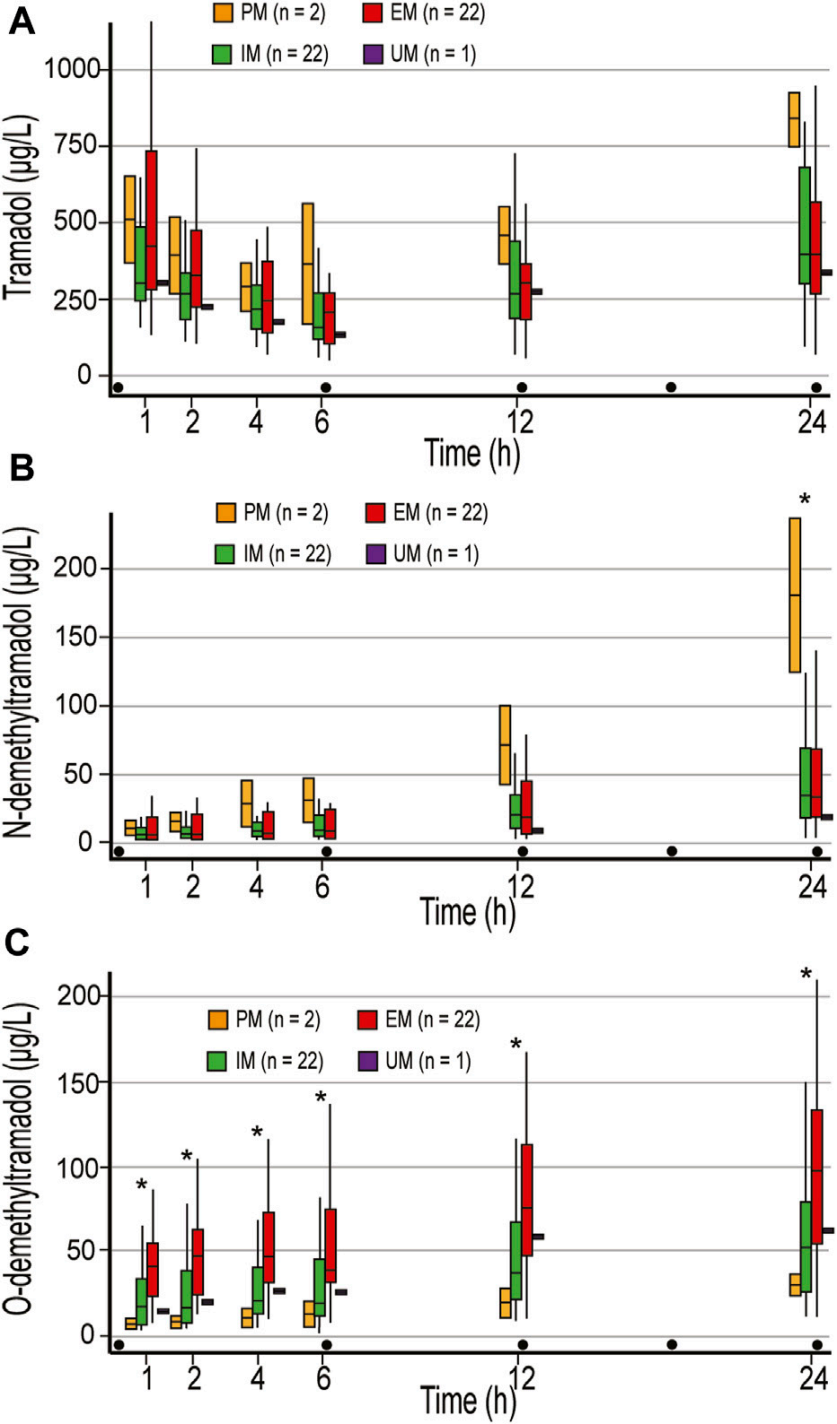
Concentrations of tramadol **(A)**, N-demethyltramadol **(B)**, and O-demethyltramadol **(C)** in the first 24 postoperative hours. Concentrations were measured 1, 2, 4 h after the first dose of 100 mg tramadol iv, and just before the second (time point 6 h), third (time point 12 h), and fifth (time point 24 h) doses of tramadol. PM, poor metabolizer; IM, intermediate metabolizer; EM, extensive metabolizer; UM, ultrafast metabolizers; Dot, tramadol 100 mg IV injections; * statistically significant differences (Mann-Whitney U test) between PM and EM/IM **(B)**, and EM and IM/PM **(C)**.

The corrected Figure 1 and its caption appear below.

The authors apologize for this error and state that this does not change the scientific conclusions of the article in any way. The original article has been updated.

