# Supplementary material for: Different Pharmacokinetics of Tramadol, O-Demethyltramadol and N-Demethyltramadol in Postoperative Surgical Patients From Those Observed in Medical Patients
Source: Front Pharmacol. 2021 Apr 15;12:656748. doi: 10.3389/fphar.2021.656748 (PMC8082457; doi:10.3389/fphar.2021.656748)

Supplementary Material

Supplementary Figure 1.

Concentrations of *O*-demethyltramadol in the NChE group (A), LChE group (B), and patients with systemic inflammation (C), with regards to metabolic phenotype. Concentrations were measured 1, 2, 4 hours after the first dose of 100 mg tramadol IV, and before the second (time point 6 h), third (time point 12 h) and fifth (time point 24 h) doses of tramadol. NChE: cholinesterase > 4244 U L^{-1}; LChE: cholinesterase ≤ 4244 U L^{-1}; IM: intermediate metabolizer; EM: extensive metabolizer; Dot: tramadol 100 mg intravenous injections; *statistically significant differences (Mann-Whitney *U* test) between EM and IM.

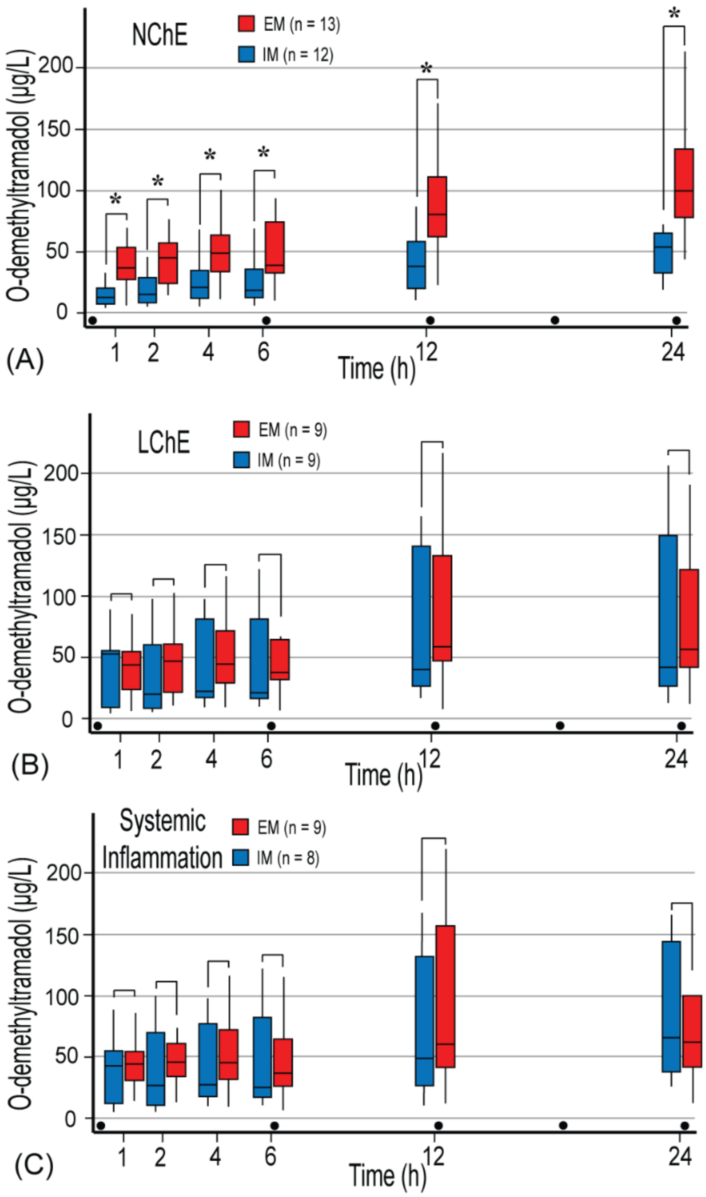

Supplement: Supplementary file 1 [file DataSheet1.pdf]
